# Methyl 2,6-diphenyl-1-*p*-tolyl-4-(*p*-tolyl­amino)-1,2,5,6-tetra­hydro­pyridine-3-carboxyl­ate

**DOI:** 10.1107/S1600536812030309

**Published:** 2012-07-07

**Authors:** K. N. Venugopala, Susanta K. Nayak, Bharti Odhav

**Affiliations:** a Department of Biotechnology and Food Technology, Durban University of Technology, Durban 4001, South Africa; b Center for Nano Science and Technology at PoliMi, Istituto Italiano di Tecnologia, Via Pascoli 70/3, 20133 Milan, Italy

## Abstract

In the title compound, C_33_H_32_N_2_O_2_, the tetra­hydro­pyridine ring adopts a boat conformation with the carbonyl group in an *s*-*cis* conformation with respect to the C=C bond of the six-membered tetra­hydro­pyridine ring. The mol­ecular conformation is stabilized by intra­molecular N—H⋯O, C—H⋯O and C—H⋯π inter­actions. Formation of centrosymmetric head-to-head dimers is observed through pairwise inter­molecular N—H⋯O hydrogen bonds. Additional weak C—H⋯O and C—H⋯π inter­actions stabilize the three-dimensional mol­ecular assembly.

## Related literature

For background to the applications of piperidines, see: Pearson *et al.* (2005 ▶); Sakai *et al.* (1986 ▶); Nayak *et al.* (2011 ▶); Mishra & Ghosh (2011 ▶). For ring-puckering parameters, see: Cremer & Pople (1975 ▶).
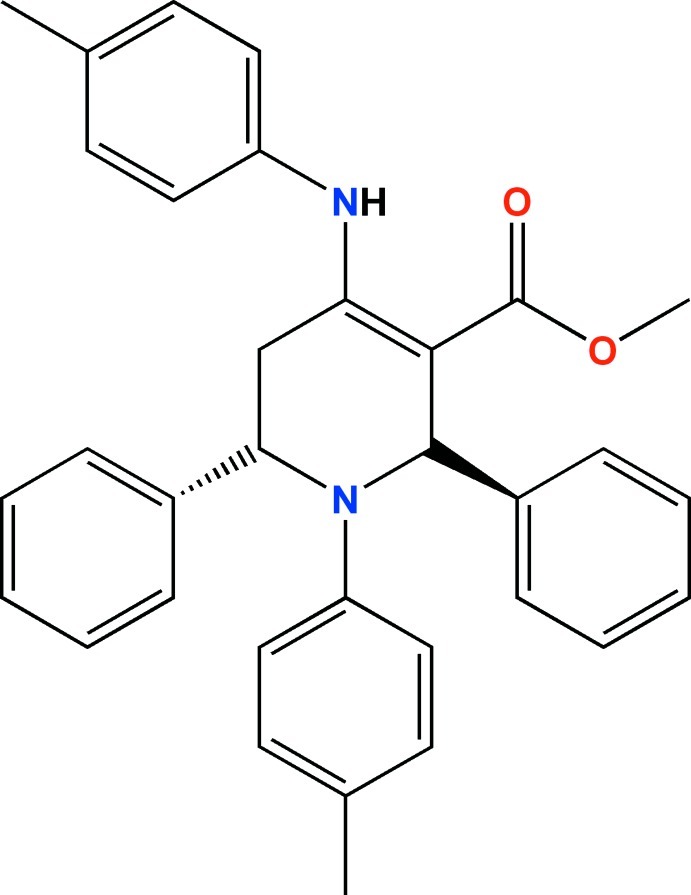


## Experimental

### 

#### Crystal data

C_33_H_32_N_2_O_2_
*M* *_r_* = 488.61
Monoclinic, 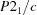

*a* = 13.3701 (11) Å
*b* = 6.1744 (5) Å
*c* = 31.797 (2) Å
β = 90.381 (2)°
*V* = 2624.8 (4) Å^3^
*Z* = 4
Mo *K*α radiation
μ = 0.08 mm^−1^
*T* = 173 K
0.29 × 0.13 × 0.05 mm

#### Data collection

Bruker Kappa DUO APEXII diffractometer
Absorption correction: multi-scan (*SADABS*; Sheldrick, 2008*b* ▶) *T*_min_ = 0.978, *T*_max_ = 0.996
16243 measured reflections
5175 independent reflections
3513 reflections with *I* > 2σ(*I*)
*R*_int_ = 0.041
Standard reflections: 0

#### Refinement

*R*[*F* ^2^ > 2σ(*F* ^2^)] = 0.044
*wR*(*F* ^2^) = 0.112
*S* = 1.03
5175 reflections
337 parameters
H-atom parameters constrained
Δρ_max_ = 0.18 e Å^−3^
Δρ_min_ = −0.26 e Å^−3^

### 

Data collection: *APEX2* (Bruker, 2008 ▶); cell refinement: *SAINT* (Bruker, 2008 ▶); data reduction: *SAINT*; program(s) used to solve structure: *SHELXS97* (Sheldrick, 2008*a* ▶); program(s) used to refine structure: *SHELXL97* (Sheldrick, 2008*a* ▶); molecular graphics: *ORTEP-3 for Windows* (Farrugia, 1997 ▶) and *Mercury* (Macrae *et al.*, 2008 ▶); software used to prepare material for publication: *PLATON* (Spek, 2009 ▶) and *PARST* (Nardelli, 1995 ▶).

## Supplementary Material

Crystal structure: contains datablock(s) global, I. DOI: 10.1107/S1600536812030309/zl2490sup1.cif

Structure factors: contains datablock(s) I. DOI: 10.1107/S1600536812030309/zl2490Isup2.hkl

Supplementary material file. DOI: 10.1107/S1600536812030309/zl2490Isup3.cml

Additional supplementary materials:  crystallographic information; 3D view; checkCIF report

## Figures and Tables

**Table 1:** Hydrogen-bond geometry (Å, °) *Cg*1, *Cg*2 and *Cg*3 are the centroids of the C29–C34, C7–C12 and C22–C27 rings, respectively.

*D*—H⋯*A*	*D*—H	H⋯*A*	*D*⋯*A*	*D*—H⋯*A*
N2—H2⋯O1	0.88	2.11	2.7343 (17)	128
C19—H19⋯O2	0.95	2.47	3.213 (3)	135
N2—H2⋯O1^i^	0.88	2.52	3.2471 (17)	140
C23—H23⋯O1^ii^	0.95	2.40	3.236 (2)	147
C24—H24⋯O1^iii^	0.95	2.58	3.363 (2)	140
C27—H27⋯*Cg*1	0.95	2.83	3.476 (2)	126
C13—H13*B*⋯*Cg*2^iv^	0.98	2.81	3.679 (2)	148
C21—H21*C*⋯*Cg*3^v^	0.98	2.97	3.487 (2)	114

Symmetry codes: (i) 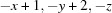; (ii) 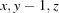; (iii) 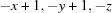; (iv) 
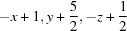
; (v) 
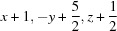
.

**Fig. 1.**
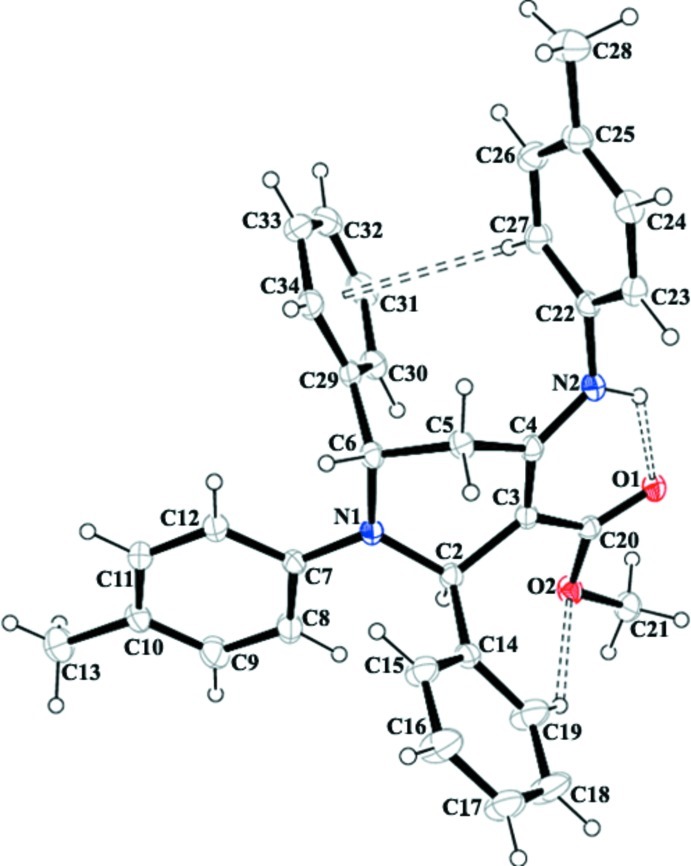
View of the molecular structure showing the atom labelling scheme with displacement ellipsoids for non-H atoms at the 30% probability level. Hydrogen atoms are shown as spheres of arbitrary radius. Dotted lines indicate intramolecular N—H···O, C—H···O, and C—H···π interactions.

**Fig. 2.**
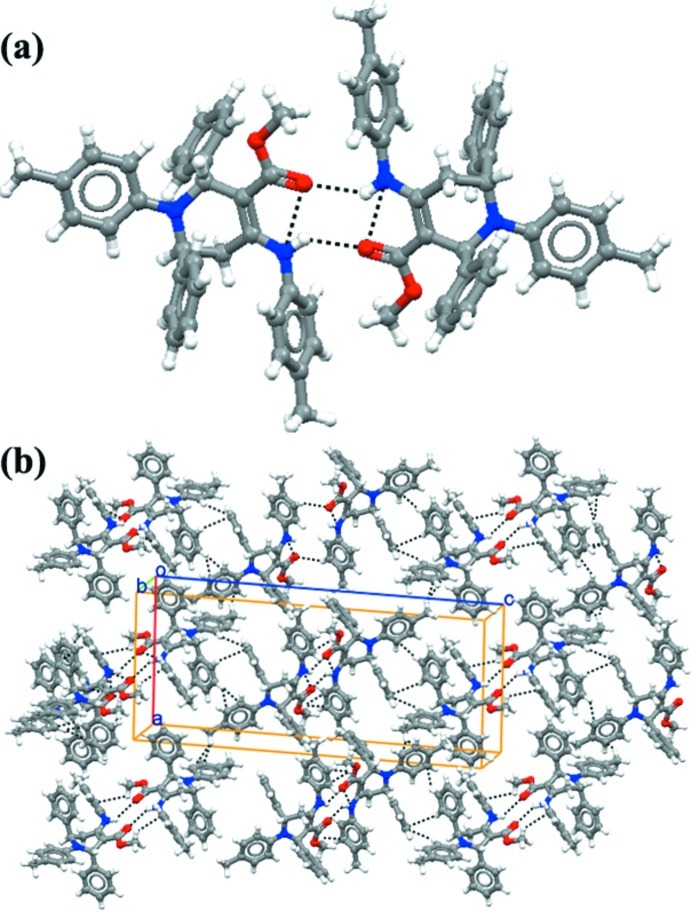
(a) Bifurcated N—H···O hydrogen bonds with both intra- and intermolecular interactions, where the later interactions lead to formation of centrosymmetric head to head dimers, (b) Weak C—H···π and C—H···O interactions further stabilize the 3-dimensional molecular assembly.

## supplementary crystallographic information

### Comment

Piperidine iminocyclitols have shown significant pharmacological results against HIV and in cancer therapy. Two deoxynojirimycin derivatives have already found clinical applications in treatment of type-II diabetes and Gaucher's disease (Pearson *et al.*, 2005). Alkaloids containing the piperidine nucleus exhibited a promising wide range of biological activities such as antimicrobial, antiparasitic, cytotoxicity, anti-inflammatory, pesticidal and anti-HIV-1 properties (Sakai *et al.*, 1986; Mishra & Ghosh, 2011). We have been investigating conformational and packing features of on tetrahyropyrimidine derivatives of compound (Nayak *et al.*, 2011). In the extension of our recent work, we have been focusing on the synthesis and biological properties of piperidine analogues. Here, we determined and analyzed the single-crystal structure of the title compound

In the title molecule, the tetrahydropyridine ring adopts a boat conformation. The Cremer & Pople (1975) parameters are Q = 0.603 (2)Å, θ = 98.16 (15)° and φ = 65.53 (15)° respectively. The carbonyl group is in a s-*cis* conformation with respect to the C═C bond of the six-membered tetrahydropyridine ring. The molecular conformation is stabilized by intramolecular N—H···O, C—H···O and C—H···π [C27—H27···*Cg*1 (centroid of C29—C34)] interactions (Fig.1, Table 1). Bifurcated N—H···O hydrogen bond patterns are observed with both intra- and intermolecular interactions. Pairwise intermolecular N—H···O interactions lead to formation of centrosymmetric head to head dimers (Fig. 2a). Further, weak C—H···O and C—H···π [C13—H13*B*···*Cg*2 (centroid of C7—C12); C21—H21*C*···*Cg*3 (centroid of C22—C27)] interactions stabilize the 3-dimensional molecular assembly (Fig. 2b).

### Experimental

To a mixture of *p*-toluidine (0.01 mol), methylacetoacetate (0.005 mmol), ZnCl_2_ (0.01 mol) and ethanol (10 ml) in a 50 ml round bottom flask was added benzaldehyde (0.01 mol). The reaction mixture was stirred at room temperature for 16 h, as monitored by TLC and then cooled to room temperature. The solid precipitate obtained was filtered and washed with aqueous ethanol to obtain a crude product (Yield 60 %; m. p. 222 (2) °C). Suitable crystals for X-ray analysis were grown from a mixture of ACN:THF (V/V;1:1) solvent by slow evaporation at room temperature.

### Refinement

All H atoms were positioned geometrically, C—H = 0.95 Å, 0.98 Å, 0.99 Å, 1.0 Å for aromatic, methyl, methylene and methine hydrogen respectively and N—H = 0.88 Å, and were refined using a riding model with *U*_iso_(H)= 1.2 *U*_eq_(C, N) for aromatic, methylene, methine and amine hydrogens, and 1.5 *U*_eq_(C) for methyl H atoms respectively.

### Crystal data

**Table:**

C_33_H_32_N_2_O_2_	*F*(000) = 1040
*M**_r_* = 488.61	*D*_x_ = 1.236 Mg m^−^^3^
Monoclinic, *P*2_1_/*c*	Melting point: 495(2) K
Hall symbol: -P 2ybc	Mo *K*α radiation, λ = 0.71073 Å
*a* = 13.3701 (11) Å	Cell parameters from 16734 reflections
*b* = 6.1744 (5) Å	θ = 1.5–26.0°
*c* = 31.797 (2) Å	µ = 0.08 mm^−^^1^
β = 90.381 (2)°	*T* = 173 K
*V* = 2624.8 (4) Å^3^	Plate, yellow
*Z* = 4	0.29 × 0.13 × 0.05 mm

### Data collection

**Table:**

Bruker Kappa DUO APEXII diffractometer	5175 independent reflections
Radiation source: fine-focus sealed tube	3513 reflections with *I* > 2σ(*I*)
Graphite monochromator	*R*_int_ = 0.041
0.5° φ scans and ω scans	θ_max_ = 26.0°, θ_min_ = 1.5°
Absorption correction: multi-scan (*SADABS*; Sheldrick, 2008*b*)	*h* = −15→16
*T*_min_ = 0.978, *T*_max_ = 0.996	*k* = −7→7
16243 measured reflections	*l* = −39→37

### Refinement

**Table:**

Refinement on *F*^2^	Primary atom site location: structure-invariant direct methods
Least-squares matrix: full	Secondary atom site location: difference Fourier map
*R*[*F*^2^ > 2σ(*F*^2^)] = 0.044	Hydrogen site location: inferred from neighbouring sites
*wR*(*F*^2^) = 0.112	H-atom parameters constrained
*S* = 1.03	*w* = 1/[σ^2^(*F*_o_^2^) + (0.0564*P*)^2^ + 0.077*P*] where *P* = (*F*_o_^2^ + 2*F*_c_^2^)/3
5175 reflections	(Δ/σ)_max_ = 0.001
337 parameters	Δρ_max_ = 0.18 e Å^−^^3^
0 restraints	Δρ_min_ = −0.26 e Å^−^^3^

### Special details

**Table:**

Geometry. All s.u.'s (except the s.u. in the dihedral angle between two l.s. planes) are estimated using the full covariance matrix. The cell s.u.'s are taken into account individually in the estimation of s.u.'s in distances, angles and torsion angles; correlations between s.u.'s in cell parameters are only used when they are defined by crystal symmetry. An approximate (isotropic) treatment of cell s.u.'s is used for estimating s.u.'s involving l.s. planes.
Refinement. Refinement of F^2^ against ALL reflections. The weighted R-factor wR and goodness of fit S are based on F^2^, conventional R-factors R are based on F, with F set to zero for negative F^2^. The threshold expression of F^2^ > 2σ(F^2^) is used only for calculating R-factors(gt) etc. and is not relevant to the choice of reflections for refinement. R-factors based on F^2^ are statistically about twice as large as those based on F, and R- factors based on ALL data will be even larger.

### Fractional atomic coordinates and isotropic or equivalent isotropic displacement parameters (Å^2^)

**Table:**

	*x*	*y*	*z*	*U*_iso_*/*U*_eq_	
O1	0.39224 (9)	1.07628 (18)	0.01596 (3)	0.0325 (3)	
O2	0.25859 (9)	1.23990 (17)	0.04498 (3)	0.0329 (3)	
N1	0.26268 (10)	0.8892 (2)	0.15100 (4)	0.0282 (3)	
N2	0.47196 (10)	0.7370 (2)	0.06014 (4)	0.0300 (3)	
H2	0.4771	0.8083	0.0363	0.036*	
C2	0.23238 (12)	0.9440 (3)	0.10784 (5)	0.0273 (4)	
H2A	0.2120	1.0998	0.1082	0.033*	
C3	0.31945 (12)	0.9271 (2)	0.07746 (5)	0.0263 (4)	
C4	0.38851 (12)	0.7657 (2)	0.08314 (5)	0.0266 (4)	
C5	0.36664 (13)	0.6094 (2)	0.11802 (5)	0.0290 (4)	
H5A	0.4256	0.5155	0.1229	0.035*	
H5B	0.3097	0.5159	0.1098	0.035*	
C6	0.34127 (12)	0.7301 (2)	0.15878 (5)	0.0272 (4)	
H6	0.3153	0.6218	0.1794	0.033*	
C7	0.21331 (12)	0.9795 (3)	0.18521 (5)	0.0274 (4)	
C8	0.14302 (14)	1.1459 (3)	0.18094 (5)	0.0408 (4)	
H8	0.1251	1.1947	0.1536	0.049*	
C9	0.09902 (15)	1.2410 (3)	0.21542 (6)	0.0479 (5)	
H9	0.0531	1.3564	0.2111	0.057*	
C10	0.11944 (14)	1.1743 (3)	0.25632 (5)	0.0410 (4)	
C11	0.18535 (14)	1.0036 (3)	0.26032 (5)	0.0400 (4)	
H11	0.1992	0.9487	0.2876	0.048*	
C12	0.23208 (13)	0.9092 (3)	0.22634 (5)	0.0360 (4)	
H12	0.2780	0.7940	0.2310	0.043*	
C13	0.07216 (17)	1.2833 (4)	0.29375 (6)	0.0606 (6)	
H13A	0.0836	1.1951	0.3190	0.091*	
H13B	0.0001	1.2988	0.2888	0.091*	
H13C	0.1021	1.4267	0.2978	0.091*	
C14	0.14036 (12)	0.8157 (3)	0.09319 (5)	0.0299 (4)	
C15	0.10575 (14)	0.6362 (3)	0.11428 (6)	0.0430 (5)	
H15	0.1383	0.5919	0.1395	0.052*	
C16	0.02464 (16)	0.5192 (3)	0.09954 (7)	0.0570 (6)	
H16	0.0031	0.3944	0.1144	0.068*	
C17	−0.02498 (16)	0.5812 (4)	0.06370 (6)	0.0561 (6)	
H17	−0.0810	0.5011	0.0537	0.067*	
C18	0.00758 (16)	0.7605 (4)	0.04252 (7)	0.0678 (7)	
H18	−0.0262	0.8057	0.0177	0.081*	
C19	0.08925 (15)	0.8762 (3)	0.05708 (6)	0.0541 (6)	
H19	0.1108	1.0002	0.0419	0.065*	
C20	0.32894 (12)	1.0818 (2)	0.04374 (5)	0.0267 (4)	
C21	0.26050 (14)	1.3986 (3)	0.01188 (5)	0.0377 (4)	
H21A	0.3238	1.4786	0.0131	0.057*	
H21B	0.2046	1.4996	0.0154	0.057*	
H21C	0.2544	1.3258	−0.0154	0.057*	
C22	0.55320 (12)	0.5962 (3)	0.07236 (5)	0.0275 (4)	
C23	0.56248 (13)	0.3923 (3)	0.05468 (5)	0.0316 (4)	
H23	0.5148	0.3442	0.0344	0.038*	
C24	0.64064 (13)	0.2587 (3)	0.06633 (5)	0.0352 (4)	
H24	0.6460	0.1193	0.0539	0.042*	
C25	0.71143 (13)	0.3237 (3)	0.09577 (5)	0.0348 (4)	
C26	0.70173 (14)	0.5291 (3)	0.11301 (5)	0.0378 (4)	
H26	0.7497	0.5776	0.1331	0.045*	
C27	0.62366 (13)	0.6650 (3)	0.10161 (5)	0.0337 (4)	
H27	0.6184	0.8049	0.1138	0.040*	
C28	0.79502 (15)	0.1731 (3)	0.10946 (7)	0.0528 (5)	
H28A	0.8358	0.2441	0.1312	0.079*	
H28B	0.8370	0.1383	0.0852	0.079*	
H28C	0.7664	0.0395	0.1209	0.079*	
C29	0.43611 (12)	0.8289 (3)	0.17719 (5)	0.0277 (4)	
C30	0.46725 (13)	1.0365 (3)	0.16685 (5)	0.0341 (4)	
H30	0.4250	1.1271	0.1504	0.041*	
C31	0.55940 (15)	1.1125 (3)	0.18032 (6)	0.0474 (5)	
H31	0.5804	1.2539	0.1726	0.057*	
C32	0.62073 (16)	0.9851 (4)	0.20476 (6)	0.0553 (6)	
H32	0.6846	1.0367	0.2134	0.066*	
C33	0.58895 (15)	0.7814 (4)	0.21667 (6)	0.0534 (6)	
H33	0.6300	0.6947	0.2344	0.064*	
C34	0.49762 (14)	0.7035 (3)	0.20290 (5)	0.0387 (4)	
H34	0.4766	0.5627	0.2111	0.046*	

### Atomic displacement parameters (Å^2^)

**Table:**

	*U*^11^	*U*^22^	*U*^33^	*U*^12^	*U*^13^	*U*^23^
O1	0.0322 (7)	0.0414 (7)	0.0238 (6)	−0.0024 (5)	0.0044 (5)	0.0027 (5)
O2	0.0369 (7)	0.0333 (6)	0.0287 (6)	0.0033 (5)	0.0057 (5)	0.0072 (5)
N1	0.0286 (8)	0.0339 (7)	0.0220 (7)	0.0044 (6)	0.0020 (6)	0.0012 (6)
N2	0.0305 (8)	0.0362 (7)	0.0234 (7)	0.0005 (6)	0.0046 (6)	0.0017 (6)
C2	0.0296 (9)	0.0290 (8)	0.0232 (8)	0.0006 (7)	0.0018 (7)	0.0027 (7)
C3	0.0269 (9)	0.0295 (8)	0.0226 (8)	−0.0039 (7)	0.0014 (6)	−0.0016 (7)
C4	0.0297 (9)	0.0281 (8)	0.0218 (8)	−0.0054 (7)	0.0006 (7)	−0.0035 (7)
C5	0.0315 (10)	0.0259 (8)	0.0299 (9)	−0.0030 (7)	0.0044 (7)	0.0007 (7)
C6	0.0319 (9)	0.0251 (8)	0.0245 (8)	0.0011 (7)	0.0045 (7)	0.0042 (7)
C7	0.0243 (9)	0.0328 (8)	0.0252 (8)	−0.0034 (7)	0.0028 (6)	−0.0015 (7)
C8	0.0444 (12)	0.0486 (11)	0.0295 (9)	0.0122 (9)	0.0045 (8)	0.0043 (8)
C9	0.0477 (12)	0.0536 (12)	0.0424 (11)	0.0182 (10)	0.0075 (9)	−0.0021 (9)
C10	0.0340 (10)	0.0565 (11)	0.0325 (9)	0.0013 (9)	0.0060 (8)	−0.0097 (9)
C11	0.0349 (11)	0.0594 (11)	0.0258 (9)	0.0009 (9)	0.0020 (7)	−0.0012 (9)
C12	0.0351 (10)	0.0446 (10)	0.0284 (9)	0.0073 (8)	0.0025 (7)	0.0010 (8)
C13	0.0560 (14)	0.0836 (16)	0.0422 (11)	0.0148 (12)	0.0087 (10)	−0.0185 (11)
C14	0.0268 (9)	0.0357 (9)	0.0273 (8)	0.0029 (7)	0.0027 (7)	0.0051 (7)
C15	0.0372 (11)	0.0499 (11)	0.0416 (10)	−0.0104 (9)	−0.0085 (8)	0.0179 (9)
C16	0.0508 (14)	0.0558 (12)	0.0640 (14)	−0.0208 (10)	−0.0177 (11)	0.0249 (11)
C17	0.0398 (12)	0.0696 (14)	0.0586 (13)	−0.0189 (11)	−0.0161 (10)	0.0163 (11)
C18	0.0499 (14)	0.0917 (17)	0.0614 (14)	−0.0244 (13)	−0.0290 (11)	0.0386 (13)
C19	0.0430 (12)	0.0645 (13)	0.0547 (12)	−0.0165 (10)	−0.0146 (10)	0.0326 (11)
C20	0.0259 (9)	0.0311 (8)	0.0230 (8)	−0.0055 (7)	−0.0022 (7)	−0.0028 (7)
C21	0.0422 (11)	0.0378 (9)	0.0330 (9)	0.0016 (8)	0.0017 (8)	0.0115 (8)
C22	0.0271 (9)	0.0309 (8)	0.0245 (8)	−0.0020 (7)	0.0051 (7)	0.0014 (7)
C23	0.0358 (10)	0.0322 (9)	0.0269 (9)	−0.0057 (8)	0.0019 (7)	−0.0018 (7)
C24	0.0416 (11)	0.0285 (8)	0.0356 (9)	−0.0011 (8)	0.0065 (8)	−0.0003 (8)
C25	0.0309 (10)	0.0366 (9)	0.0369 (9)	−0.0015 (8)	0.0056 (8)	0.0093 (8)
C26	0.0352 (11)	0.0418 (10)	0.0365 (10)	−0.0067 (8)	−0.0057 (8)	0.0005 (8)
C27	0.0384 (11)	0.0323 (9)	0.0305 (9)	−0.0025 (8)	−0.0007 (8)	−0.0057 (7)
C28	0.0395 (12)	0.0491 (11)	0.0697 (14)	0.0027 (9)	−0.0011 (10)	0.0114 (11)
C29	0.0301 (9)	0.0328 (8)	0.0204 (8)	0.0037 (7)	0.0043 (7)	−0.0019 (7)
C30	0.0369 (10)	0.0326 (9)	0.0330 (9)	0.0000 (8)	0.0053 (8)	−0.0043 (8)
C31	0.0419 (12)	0.0509 (11)	0.0495 (12)	−0.0120 (10)	0.0110 (10)	−0.0199 (10)
C32	0.0337 (12)	0.0867 (16)	0.0457 (12)	−0.0020 (11)	0.0016 (9)	−0.0338 (12)
C33	0.0404 (12)	0.0858 (16)	0.0339 (10)	0.0226 (12)	−0.0071 (9)	−0.0114 (11)
C34	0.0423 (11)	0.0455 (10)	0.0282 (9)	0.0132 (9)	0.0058 (8)	0.0018 (8)

### Geometric parameters (Å, º)

**Table:**

O1—C20	1.2277 (18)	C15—H15	0.9500
O2—C20	1.3562 (19)	C16—C17	1.369 (3)
O2—C21	1.4385 (18)	C16—H16	0.9500
N1—C7	1.393 (2)	C17—C18	1.368 (3)
N1—C6	1.458 (2)	C17—H17	0.9500
N1—C2	1.4679 (19)	C18—C19	1.382 (3)
N2—C4	1.3498 (19)	C18—H18	0.9500
N2—C22	1.443 (2)	C19—H19	0.9500
N2—H2	0.8800	C21—H21A	0.9800
C2—C3	1.521 (2)	C21—H21B	0.9800
C2—C14	1.533 (2)	C21—H21C	0.9800
C2—H2A	1.0000	C22—C23	1.384 (2)
C3—C4	1.370 (2)	C22—C27	1.386 (2)
C3—C20	1.442 (2)	C23—C24	1.380 (2)
C4—C5	1.500 (2)	C23—H23	0.9500
C5—C6	1.535 (2)	C24—C25	1.386 (2)
C5—H5A	0.9900	C24—H24	0.9500
C5—H5B	0.9900	C25—C26	1.388 (2)
C6—C29	1.521 (2)	C25—C28	1.516 (2)
C6—H6	1.0000	C26—C27	1.385 (2)
C7—C8	1.398 (2)	C26—H26	0.9500
C7—C12	1.399 (2)	C27—H27	0.9500
C8—C9	1.379 (2)	C28—H28A	0.9800
C8—H8	0.9500	C28—H28B	0.9800
C9—C10	1.389 (2)	C28—H28C	0.9800
C9—H9	0.9500	C29—C30	1.388 (2)
C10—C11	1.379 (3)	C29—C34	1.391 (2)
C10—C13	1.510 (2)	C30—C31	1.384 (3)
C11—C12	1.381 (2)	C30—H30	0.9500
C11—H11	0.9500	C31—C32	1.373 (3)
C12—H12	0.9500	C31—H31	0.9500
C13—H13A	0.9800	C32—C33	1.381 (3)
C13—H13B	0.9800	C32—H32	0.9500
C13—H13C	0.9800	C33—C34	1.381 (3)
C14—C15	1.377 (2)	C33—H33	0.9500
C14—C19	1.384 (2)	C34—H34	0.9500
C15—C16	1.382 (3)		
			
C20—O2—C21	116.96 (12)	C17—C16—H16	119.6
C7—N1—C6	118.83 (12)	C15—C16—H16	119.6
C7—N1—C2	120.57 (13)	C18—C17—C16	118.79 (19)
C6—N1—C2	120.52 (12)	C18—C17—H17	120.6
C4—N2—C22	123.83 (13)	C16—C17—H17	120.6
C4—N2—H2	118.1	C17—C18—C19	120.44 (18)
C22—N2—H2	118.1	C17—C18—H18	119.8
N1—C2—C3	111.74 (13)	C19—C18—H18	119.8
N1—C2—C14	112.39 (13)	C18—C19—C14	121.54 (18)
C3—C2—C14	112.80 (13)	C18—C19—H19	119.2
N1—C2—H2A	106.5	C14—C19—H19	119.2
C3—C2—H2A	106.5	O1—C20—O2	121.50 (14)
C14—C2—H2A	106.5	O1—C20—C3	125.50 (15)
C4—C3—C20	121.17 (14)	O2—C20—C3	113.00 (13)
C4—C3—C2	118.95 (13)	O2—C21—H21A	109.5
C20—C3—C2	119.88 (14)	O2—C21—H21B	109.5
N2—C4—C3	125.65 (14)	H21A—C21—H21B	109.5
N2—C4—C5	118.82 (14)	O2—C21—H21C	109.5
C3—C4—C5	115.53 (14)	H21A—C21—H21C	109.5
C4—C5—C6	110.94 (12)	H21B—C21—H21C	109.5
C4—C5—H5A	109.5	C23—C22—C27	119.24 (15)
C6—C5—H5A	109.5	C23—C22—N2	120.53 (14)
C4—C5—H5B	109.5	C27—C22—N2	120.23 (14)
C6—C5—H5B	109.5	C24—C23—C22	120.29 (16)
H5A—C5—H5B	108.0	C24—C23—H23	119.9
N1—C6—C29	113.15 (13)	C22—C23—H23	119.9
N1—C6—C5	110.29 (12)	C23—C24—C25	121.36 (16)
C29—C6—C5	109.36 (13)	C23—C24—H24	119.3
N1—C6—H6	108.0	C25—C24—H24	119.3
C29—C6—H6	108.0	C24—C25—C26	117.78 (16)
C5—C6—H6	108.0	C24—C25—C28	121.00 (16)
N1—C7—C8	122.69 (14)	C26—C25—C28	121.20 (17)
N1—C7—C12	121.50 (15)	C27—C26—C25	121.48 (16)
C8—C7—C12	115.80 (15)	C27—C26—H26	119.3
C9—C8—C7	121.71 (16)	C25—C26—H26	119.3
C9—C8—H8	119.1	C26—C27—C22	119.84 (16)
C7—C8—H8	119.1	C26—C27—H27	120.1
C8—C9—C10	122.38 (18)	C22—C27—H27	120.1
C8—C9—H9	118.8	C25—C28—H28A	109.5
C10—C9—H9	118.8	C25—C28—H28B	109.5
C11—C10—C9	115.75 (16)	H28A—C28—H28B	109.5
C11—C10—C13	122.59 (17)	C25—C28—H28C	109.5
C9—C10—C13	121.65 (18)	H28A—C28—H28C	109.5
C10—C11—C12	122.88 (17)	H28B—C28—H28C	109.5
C10—C11—H11	118.6	C30—C29—C34	118.43 (16)
C12—C11—H11	118.6	C30—C29—C6	122.00 (14)
C11—C12—C7	121.39 (16)	C34—C29—C6	119.41 (15)
C11—C12—H12	119.3	C31—C30—C29	120.52 (17)
C7—C12—H12	119.3	C31—C30—H30	119.7
C10—C13—H13A	109.5	C29—C30—H30	119.7
C10—C13—H13B	109.5	C32—C31—C30	120.53 (19)
H13A—C13—H13B	109.5	C32—C31—H31	119.7
C10—C13—H13C	109.5	C30—C31—H31	119.7
H13A—C13—H13C	109.5	C31—C32—C33	119.53 (19)
H13B—C13—H13C	109.5	C31—C32—H32	120.2
C15—C14—C19	117.11 (16)	C33—C32—H32	120.2
C15—C14—C2	122.64 (14)	C34—C33—C32	120.22 (19)
C19—C14—C2	120.24 (15)	C34—C33—H33	119.9
C14—C15—C16	121.39 (17)	C32—C33—H33	119.9
C14—C15—H15	119.3	C33—C34—C29	120.68 (18)
C16—C15—H15	119.3	C33—C34—H34	119.7
C17—C16—C15	120.71 (18)	C29—C34—H34	119.7
			
C7—N1—C2—C3	151.62 (14)	C3—C2—C14—C19	−65.7 (2)
C6—N1—C2—C3	−31.79 (19)	C19—C14—C15—C16	1.2 (3)
C7—N1—C2—C14	−80.38 (18)	C2—C14—C15—C16	−177.72 (19)
C6—N1—C2—C14	96.20 (17)	C14—C15—C16—C17	−1.2 (3)
N1—C2—C3—C4	36.66 (19)	C15—C16—C17—C18	0.4 (4)
C14—C2—C3—C4	−91.11 (17)	C16—C17—C18—C19	0.2 (4)
N1—C2—C3—C20	−142.40 (14)	C17—C18—C19—C14	−0.1 (4)
C14—C2—C3—C20	89.83 (17)	C15—C14—C19—C18	−0.6 (3)
C22—N2—C4—C3	166.10 (15)	C2—C14—C19—C18	178.4 (2)
C22—N2—C4—C5	−14.4 (2)	C21—O2—C20—O1	1.0 (2)
C20—C3—C4—N2	2.5 (2)	C21—O2—C20—C3	−178.39 (13)
C2—C3—C4—N2	−176.52 (14)	C4—C3—C20—O1	5.7 (2)
C20—C3—C4—C5	−176.98 (13)	C2—C3—C20—O1	−175.28 (15)
C2—C3—C4—C5	4.0 (2)	C4—C3—C20—O2	−174.94 (13)
N2—C4—C5—C6	131.03 (15)	C2—C3—C20—O2	4.1 (2)
C3—C4—C5—C6	−49.43 (18)	C4—N2—C22—C23	100.73 (18)
C7—N1—C6—C29	−71.72 (18)	C4—N2—C22—C27	−80.1 (2)
C2—N1—C6—C29	111.64 (15)	C27—C22—C23—C24	0.5 (2)
C7—N1—C6—C5	165.43 (13)	N2—C22—C23—C24	179.67 (15)
C2—N1—C6—C5	−11.21 (19)	C22—C23—C24—C25	0.0 (2)
C4—C5—C6—N1	52.06 (17)	C23—C24—C25—C26	−0.5 (2)
C4—C5—C6—C29	−72.98 (16)	C23—C24—C25—C28	177.92 (16)
C6—N1—C7—C8	174.53 (15)	C24—C25—C26—C27	0.5 (3)
C2—N1—C7—C8	−8.8 (2)	C28—C25—C26—C27	−177.91 (17)
C6—N1—C7—C12	−5.0 (2)	C25—C26—C27—C22	0.0 (3)
C2—N1—C7—C12	171.68 (15)	C23—C22—C27—C26	−0.5 (2)
N1—C7—C8—C9	−176.44 (17)	N2—C22—C27—C26	−179.67 (15)
C12—C7—C8—C9	3.1 (3)	N1—C6—C29—C30	−33.1 (2)
C7—C8—C9—C10	−1.8 (3)	C5—C6—C29—C30	90.31 (17)
C8—C9—C10—C11	−1.1 (3)	N1—C6—C29—C34	151.50 (14)
C8—C9—C10—C13	178.74 (19)	C5—C6—C29—C34	−85.13 (17)
C9—C10—C11—C12	2.7 (3)	C34—C29—C30—C31	2.9 (2)
C13—C10—C11—C12	−177.19 (19)	C6—C29—C30—C31	−172.61 (15)
C10—C11—C12—C7	−1.3 (3)	C29—C30—C31—C32	−1.2 (3)
N1—C7—C12—C11	177.96 (16)	C30—C31—C32—C33	−1.4 (3)
C8—C7—C12—C11	−1.6 (3)	C31—C32—C33—C34	2.2 (3)
N1—C2—C14—C15	−14.2 (2)	C32—C33—C34—C29	−0.5 (3)
C3—C2—C14—C15	113.24 (18)	C30—C29—C34—C33	−2.1 (2)
N1—C2—C14—C19	166.88 (16)	C6—C29—C34—C33	173.55 (15)

### Hydrogen-bond geometry (Å, º)

*Cg*1, *Cg*2 and *Cg*3 are the centroids of the C29–C34, C7–C12 and C29–C34 rings, respectively. [Cg3 and Cg1 are the same ring (information taken from Comment section)?]

**Table:**

*D*—H···*A*	*D*—H	H···*A*	*D*···*A*	*D*—H···*A*
N2—H2···O1	0.88	2.11	2.7343 (17)	128
C19—H19···O2	0.95	2.47	3.213 (3)	135
N2—H2···O1^i^	0.88	2.52	3.2471 (17)	140
C23—H23···O1^ii^	0.95	2.40	3.236 (2)	147
C24—H24···O1^iii^	0.95	2.58	3.363 (2)	140
C27—H27···*Cg*1	0.95	2.83	3.476 (2)	126
C13—H13*B*···*Cg*2^iv^	0.98	2.81	3.679 (2)	148
C21—H21*C*···*Cg*3^v^	0.98	2.97	3.487 (2)	114

Symmetry codes: (i) −*x*+1, −*y*+2, −*z*; (ii) *x*, *y*−1, *z*; (iii) −*x*+1, −*y*+1, −*z*; (iv) −*x*+1, *y*+5/2, −*z*+1/2; (v) *x*+1, −*y*+5/2, *z*+1/2.
